# Characterization of a Novel *BCHE* “Silent” Allele: Point Mutation (p.Val204Asp) Causes Loss of Activity and Prolonged Apnea with Suxamethonium

**DOI:** 10.1371/journal.pone.0101552

**Published:** 2014-07-23

**Authors:** Herve Delacour, Sofya Lushchekina, Isabelle Mabboux, Aurore Bousquet, Franck Ceppa, Lawrence M. Schopfer, Oksana Lockridge, Patrick Masson

**Affiliations:** 1 Bégin Military Teaching Hospital, Department of Biology, Unit of Human Genetics, Saint Mandé, France; 2 Modeling of Biomolecules Lab., N.M. Emanuel Institute of Biochemical Physics, Russian Academy of Sciences, Moscow, Russia; 3 Val de Grace Military Medical School, Paris, France; 4 Eppley Institute, University of Nebraska Medical Center, Omaha, Nebraska, United States of America; 5 DYNAMOP, Institut de Biologie Structurale, Grenoble, France; University Paris Diderot-Paris 7, France

## Abstract

Butyrylcholinesterase deficiency is characterized by prolonged apnea after the use of muscle relaxants (suxamethonium or mivacurium) in patients who have mutations in the *BCHE* gene. Here, we report a case of prolonged neuromuscular block after administration of suxamethonium leading to the discovery of a novel *BCHE* variant (c.695T>A, p.Val204Asp). Inhibition studies, kinetic analysis and molecular dynamics were undertaken to understand how this mutation disrupts the catalytic triad and determines a “silent” phenotype. Low activity of patient plasma butyrylcholinesterase with butyrylthiocholine (BTC) and benzoylcholine, and values of dibucaine and fluoride numbers fit with heterozygous atypical silent genotype. Electrophoretic analysis of plasma BChE of the proband and his mother showed that patient has a reduced amount of tetrameric enzyme in plasma and that minor fast-moving BChE components: monomer, dimer, and monomer-albumin conjugate are missing. Kinetic analysis showed that the p.Val204Asp/p.Asp70Gly-p.Ala539Thr BChE displays a pure Michaelian behavior with BTC as the substrate. Both catalytic parameters K_m_ = 265 µM for BTC, two times higher than that of the atypical enzyme, and a low V_max_ are consistent with the absence of activity against suxamethonium. Molecular dynamic (MD) simulations showed that the overall effect of the mutation p.Val204Asp is disruption of hydrogen bonding between Gln223 and Glu441, leading Ser198 and His438 to move away from each other with subsequent disruption of the catalytic triad functionality regardless of the type of substrate. MD also showed that the enzyme volume is increased, suggesting a pre-denaturation state. This fits with the reduced concentration of p.Ala204Asp/p.Asp70Gly-p.Ala539Thr tetrameric enzyme in the plasma and non-detectable fast moving-bands on electrophoresis gels.

## Introduction

Butyrylcholinesterase (EC 3.1.1.8; BChE) also known as pseudocholinesterase, is the sister enzyme of acetylcholinesterase (EC 3.1.1.7; AChE). It is present in most tissues and in human plasma at a concentration about 50 nM. Though BChE lacks obvious physiological functions, it is of toxicological and pharmacological importance in detoxifying or catabolising ester-containing drugs [1]. Furthermore, individuals deficient in BChE appear asymptomatic, apart from a heightened sensitivity to the muscle relaxants suxamethonium and mivacurium, two BChE substrates used as myorelaxant [2]. In patients with usual BChE levels, these drugs are rapidly hydrolysed in plasma and their duration of action is short (<10 min). BChE deficiency results in slower hydrolysis of these drugs and, consequently, a prolonged neuromuscular block, leading to apnea. Prolonged neuromuscular block occurs with BChE deficiencies of marked severity (impairment >70%). Although many acquired conditions may affect BChE activity (liver or renal diseases, malnutrition, pregnancy, malignancy), BChE deficiency is mainly due to mutations in the *BCHE* gene (MIM 177400) [2].

Prolonged apnea following injection of succinylcholine was first described in 1953 [3]. The genetic variation of BChE deficiency was described by Kalow and Genest in 1957 and is said to be a cornerstone in pharmacogenetics/pharmacogenomics [4]. The human *BCHE* gene is located on chromosome *3q26.1*, contains 3 coding exons, and spans approximately 64 kb. Genetically inherited BChE deficiency shows autosomal recessive inheritance. It has been estimated that almost 24% of the human population carries at least one variant *BCHE* allele [5]. Currently, close to 70 natural mutations have been documented in human *BCHE*. Most of them have an adverse effect on BChE activity. This may occur either by deleterious effects of point mutations on catalytic functioning, or by point mutations that affect protein expression, which may result in an absence of BChE altogether [2]. Although the majority of these mutations are rare, two of them, the atypical variant (c.293A>G, p.Asp70Gly, rs1799807) and the K-variant (c.1699G>A, p.Ala539Tyr, rs1803274), are relatively common, homozygotes occurring at 1∶3,000 and 1∶100 in the Caucasian population respectively.

Here we report a case of prolonged neuromuscular block after administration of suxamethonium leading to the discovery of a novel *BCHE* variant. DNA sequencing showed that this variant results from a point mutation c.695T>A (p.Val204Asp). Inhibition studies, kinetic analysis and molecular dynamics were undertaken to understand how this mutation determines the “silent” phenotype.

## Subjects and Methods

### Patients

The patient is a one-month old infant boy (subject II-1), who presented a prolonged neuromuscular block (16 hours) after administration of a single dose of succinylcholine (2 mg/kg) for pyloric stenosis surgery. His parents have no medical or surgical history. Biological investigations mentioned below were performed on plasma from the patient and his parents. The parents signed an informed consent to participate in the genetic study, which was approved by the Research and Ethics Committee of the Begin Military Teaching Hospital.

### Reference plasma samples

Three reference plasma samples of phenotype UU, UA and AS (DNA sequence for the silent BChE unknown) from O. Lockridge’s plasma sample collection were used for control of catalytic and inhibition properties and electrophoresis of plasma BChE samples.

### Chemicals

Butyrylthiocholine iodide (BTC), Benzoylcholine chloride (BzCh) and dibucaine chloride were purchased from Sigma (St Louis, Missouri, United States of America). Succinyldithiocholine was custom synthesized by Molecular Probes Inc. Other chemicals were of biochemical grade.

### BChE activity

Total plasma BChE activity was measured with butyrylthiocholine iodide (BTC) as substrate on a Cobas® 6000 system (Roche Diagnostics) according to the method described by the manufacturer ([BTC] = 7.56 mM at pH = 7.7 and 37°C). Under these conditions the reference interval for BChE activity in healthy individuals was 5,320–12,920 IU/L for men, 4,260–11,250 IU/L for women under 39-years, and 2,260–6,460 IU/L for infants [6].

Activity of BChE in plasma or serum was also measured in the laboratory using a single beam spectrophotometer. Two substrates were used: BTC and BzCh. Activity with BTC was determined using Ellman’s method in 0.1 M phosphate buffer pH 7.0 at 25°C [7]. Plasma samples (10 µL) were preincubated for 5 min with the chromogenic reagent 20 mM DTNB (50 µL) in 1,275 µL of the buffer solution prior to assay to neutralize plasma-containing free thiol compounds (under these conditions, free-thiol-containing molecules present in plasma samples were neutralized in less that 3 min). After adding 1 mM BTC, assays were recorded at 412 nm taking ε_412_ = 16,360 M^−1 ^cm^−1^. Activity with BzCh was determined according to Kalow and Genest by recording decrease in absorbance at 240 nm (Δε = 6,700 M^−1^.cm^−1^) of 50 µM BzCh in 67 mM phosphate buffer pH 7.4 at 25°C [4]. For both assays, one unit (U) of enzyme activity is defined as the amount of enzyme that hydrolyzes one µmol of substrate per minute.

### Genotyping and mutational analysis

Blood samples were collected from the patient and his parents. Genomic DNA was extracted from peripheral blood leucocytes with use of the QIAamp Blood-DNA mini reagent set (Qiagen) according to the manufacturer’s instructions. DNA was eluted in 100 µL of water in the final step and stored at −20°C until required. Sequencing of the coding exons and exon-intron boundaries of the *BCHE* gene was performed after PCR amplification of genomic DNA, with specific primers (Table 1). All of the coding exons and the flanking introns of the *BCHE* gene were amplified by PCR using the following conditions: 1 cycle of 95°C for 10 min; 40 cycles of denaturation at 95°C for 30 s, annealing at 56°C for 30 s, and an extension at 72°C for 40 s; 1 cycle of 72°C for 7 min. The reaction mixture of final volume 25 µL contained 100 ng DNA template, 1×PCR Buffer (Invitrogen), 2.0 mmol MgCl_2_, 0.2 mmol dNTP, 1 µmol of each primer, and 0,625 U Taq Gold DNA polymerase. PCR products were purified with use of the High Pure PCR Product Purification Kit (Roche Diagnostics) according to the manufacturer’s instructions. Both strands were sequenced using amplification primers as sequencing primers and BigDyeDeoxy terminator cycle sequencing according to the manufacturer’s instruction (Beckman Coulter). Purified sequencing fragments were separated by capillary electrophoresis and detected by laser-induced fluorescence on a Beckman Ceq 8000 (Beckman Coulter). Polyphen [8] and Mutation Taster [9] were used to perform *in silico* prediction of the impact of nucleotide changes on the protein structure and function.

**Table 1:** Oligonucleotide primers used in PCR for the amplification of the *BCHE* gene.

Primer name	Sequence (5′-3′)	Tm
2–1F	GGCCTTTACAGAAGCAGGTTT	59.8
2–1R	TTTTTGGTTTAGGTGCTGGAA	59.6
2–2F	CGGTAACAGCCTTTCTTGGA	60.2
2–2R	AGCTGCTCCTGCACTTTCTC	59.9
2–3F	GGATTCCAGCACCTAAACCA	59.9
2–3R	CCCATAGGGGACAACAAATG	60.0
2–4F	GGTTGCTCTAGAGAGAATGAGACTG	60.1
2–4R	GTTTGGAGGATCGGTGTTCA	60.9
2–5F	CCAGGAGTGAGTGAGTTTGGA	60.3
2–5R	AAAAATAAAACCAGCTTTGGTACA	58.3
3F	TTCTTTAAAGCTCTTGTGAACAGTG	59.2
3R	CACCGTGCCTTGGAGAGTAT	60.1
4F	GAGAAAATGGCTTTTGTATTCG	58.0
4R	CGTTCTAGCCATTTGGGTTT	59.1

### Inhibition studies

Inhibition numbers (i.e. the dibucaine number and the fluoride number) for plasma BChE samples were determined according to classical procedures using BzCh as the substrate in 67 mM phosphate buffer pH 7.4 at 25°C. An inhibition number is defined as the percentage of inhibition of hydrolysis of 50 µM BzCh by a fixed concentration of inhibitor. The dibucaine number (DN) was determined using 10 µM dibucaine according to Kalow and Genest [4]. The fluoride number (FN) was determined using 50 µM sodium fluoride according to Harris and Whittaker [10].

### Determination of catalytic parameters

Catalytic parameters for hydrolysis of BTC were determined in 0.1 M phosphate buffer pH 7.0 at 25°C. Measurements were performed using Ellman’s method [7]. Hydrolysis rates were recorded for 2 min after preincubation of plasma samples in the presence of DTNB as mentioned above. Wide ranges of substrate concentrations were used: from 10 µM to 50 mM for BTC. Spontaneous hydrolysis of substrates was recorded and subtracted for each enzymatic measurement at corresponding substrate concentrations.

**Figure 1:**
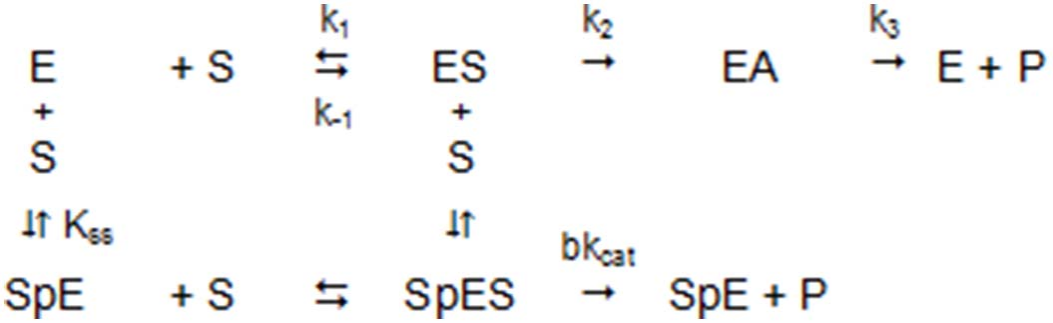
Model of BChE catalyzed-hydrolysis of substrates.

BChE catalyzed-hydrolysis of substrates can be described by the model (Fig. 1) and by Eq 1 [11]:
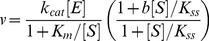
(1)


At low substrate concentration, hydrolysis kinetics for all substrates follow the simple boxed reaction scheme that corresponds to the simple Michaelis-Menten mechanism in which the Michaelis constant, K_m_, is described by Eq 2:
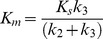
(2)with K_s_ = k_-1_/k_1_, the dissociation of the ES complex, k_2_ and k_3_ the rate constant for acylation (EA) and deacylation, respectively. The catalytic constant k_cat_ is:
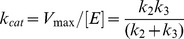
(3)with V_max_, the maximum velocity, and [E], the enzyme active site concentration.

At high concentration, for positively charged substrates like BTC a second substrate molecule binds to the peripheral anionic site (PAS, p) of wild-type enzyme (Usual): SpE, and the ternary complex S_P_ES makes products. K_ss_ is the dissociation constant of this ternary complex. As a consequence, the kinetics deviate from the Michaelian model; there is either activation by excess substrate, e.g. with BTC, or inhibition by excess substrate, e.g. with BzCh. In Eq 1, the b factor refers to the effect of the second substrate molecule on the k_cat_. If b>1, there is activation; if b<1, there is inhibition. If there is no binding on the PAS, b = 1, and Eq 1 reduces to the simple Michaelis-Menten equation.

Catalytic parameters were determined by non-linear fitting of substrate-dependent hydrolysis rate curves. Because BChE has a non-Michaelian behavior with BTC as substrate, Eq 1 was used to describe hydrolysis of BTC. On the other hand, owing to the structure of SCdTC which contains 2 thiocholine groups (one binds on the catalytic active center and the other binds to the PAS) the catalytic behavior is Michaelian, and Eq 1 reduces to Eq 3 [12].

### Nondenaturing gradient gel electrophoresis

Nondenaturing gradient gel (4–30%) electrophoresis of plasma samples (5 µL/lane) was carried out as described [13]. BChE active bands were stained using the methods of Karnovsky and Roots [14] with BTC as the substrate as described elsewhere [13].

### Micropurification of plasma BChE and denaturing gel electrophoresis

BChE was immunopurified from 0.5 ml plasma by binding to monoclonal mAb2 immobilized on Sepharose 4B [15]. Activity assays showed that 97% of the BChE activity had disappeared from plasma following overnight incubation with mAb2-Sepharose. Beads (0.04 mL) were washed with phosphate buffered saline, and desalted by washing with water. Beads were transferred to 0.45 µm Durapore centrifugal filters. BChE protein was released from the beads with 80 µL of 0.4 M acetic acid pH 2.6. The filtered acid extract was dried in a vacuum centrifuge, dissolved in 35 µL of SDS/dithiothreitol loading buffer, heated in a boiling water bath for 3 min and loaded on an SDS gel. Denaturing gel electrophoresis in SDS in the presence of dithiothreitol was carried out according to Laemli [16] on 4–30% gradient polyacrylamide gels. Protein bands were stained with Coomassie Brilliant Blue.

### Molecular dynamics study

Model structures for wild type (usual BChE) and p.Val204Asp mutant were prepared from the X-ray coordinates of PDB ID 1P0I [17] as described in our previous studies [18], [19]. For p.Val204Asp mutant of BChE, the Val204 side chain was manually converted to aspartic acid and its side chain was minimized with other protein atoms fixed. Minimization and MD simulations were performed with the NAMD 2.9 program [20] and the CHARMM36 force field [21] on the Lomonosov Moscow State University supercomputer [22]. After solvation and addition of ions, protein structures were saturated with water molecules and water box equilibrating runs were performed as described in [18]. This was followed by a full dynamic simulation of each system performed for 100 ns under periodic boundary conditions at a constant temperature of 298 K and constant pressure 1 atm. Structural analysis of the obtained MD trajectories was performed with ProDy [23] and VMD [24] software packages.

## Results and Discussion

The crystal structure of human BChE was described in 2003 [17]. The active, or acylation site (Ser198…His438…Glu325 triad) lies near the base of a gorge, approximately 20 Å from the surface. An adjacent site, the choline-binding site, also called the anionic site (Trp82…Tyr128…Phe329), is responsible for binding the choline moiety of the substrate, and positioning the ester bond for nucleophilic attack by the serine of the active site. Two additional structures, also located near the base of the gorge, are the oxyanion hole (Gly116…Gly117…Ala199) and the acyl pocket (Trp231…Leu286…Val288), which stabilize the substrate during hydrolysis. A peripheral anionic site (Asp70…Tyr332) is involved in the initial binding of the substrate and in activation control.

Various mutations affect BChE activity. In some cases, residues involved in the catalytic process are mutated: e.g. Ser198 of the active site, Tyr128 of the choline binding site, or Asp70 of the peripheral site. The first two mutations result in a silent enzyme, while the last one causes a reduction in substrate affinity and an abolition of substrate activation. Other mutated residues fall within conserved sequences or motifs that are distant from the active site.

**Figure 2:**
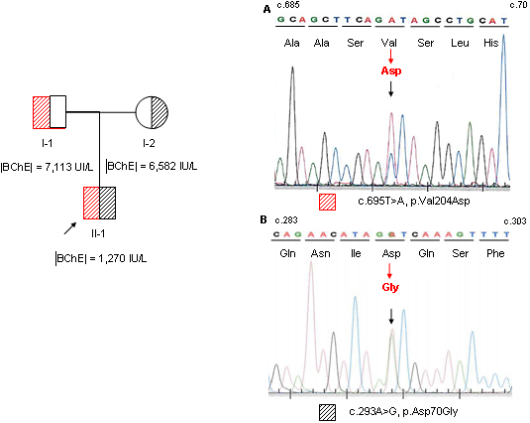
Pedigree of the family with BChE activities and genotypes. BChE activities were measured on Cobas® 6000 system. Results of genotyping are given as electropherograms. Sequence analyses reveal (A) an heterozygous mutation c.695T>A (p.Val204Asp). This mutation is observed in the proband and in patient I-1; (B) an heterozygous mutation c.293A>G (p.Asp70Gly, atypical variant). This mutation is observed in the proband and in patient I-2 and (C) an heterozygous mutation c.1699G>A (p.Ala539Thr, Kalow variant, electrophorogram not shown). This mutation is observed in the proband and in patient I-2.

Here we report a novel *BCHE* variant in a patient presenting a marked deficiency in BChE activity (|BChE| = 1,270 IU/L) (Fig. 2). Sequencing of the whole coding region of *BCHE* revealed that this patient harbored three point mutations in a compound heterozygous state. One was the well-known atypical variant (c.293A>G, p.Asp70Gly). The second one was the Kalow-variant (c.1699G>A, p.Ala539Thr). The last one, which was not previously described nor was included in any mutation databases, introduced a mis-sense mutation at amino acid residue 204 (c.695T>A, p.Val204Asp, GenBank Accession #KJ513459) (Fig. 2). It involves a residue highly conserved across species (Table 2). *In silico* predictions identified this mutation as potentially deleterious: i.e. PolyPhen-2 score of 1.0 (sensitivity: 0.00, specificity: 1.00), and Mutation Taster score of 152. This mutation was observed in the patient’s father (patient I-1) at heterozygous state, whereas the mother (patient I-2) was compound heterozygote for the atypical variant and the Kalow variant.

**Table 2:** Evolutionary conservation of the region of BChE including in the variant site p.Val204Asp.

Class	Species	Sequence	Swissprot accession #
Mammalia	*H. sapiens*	TLFGESAGAAS**V**SLHLLSPG	P06276
	*B. taurus*	TLFGESAGAAS**V**SLHLLSPE	P32749
	*O. cuniculus*	TLFGESAGAAS**V**SLHLLSPR	P21927
	*F. catus*	TLFGESAGAGS**V**SLHLLSPR	O62760
	*C. familiaris*	TLFGESAGAGS**V**GLHLLSPR	F1PEN8
Aves	*G. gallus*	TIFGESAGSAS**V**SYHILSPK	Q90ZK8
Amphibia	*X. tropicalis*	TIFGHSAGAAS**V**GFHLISTK	F7BPW9
Osteichthyes	*G. aculeatus*	TLFGESAGSAS**V**GFHLLSPG	G3NW96

Mutations near residue Val204 have previously been described: p.Ala199Val [25], p.Ala201Thr [26] and p.Ser203Pro [27]. These mutations result in a silent enzyme. However, the mechanisms by which these mutations determine the silent phenotype were not established.

In the present work kinetic analysis, inhibition studies and molecular dynamics studies have led to an understanding of how mutation p.Val204Asp disrupts the catalytic triad and determines the “silent” phenotype.

Results of phenotyping are in Table 3. Results are consistent with reported values of activity, DN and FN for heterozygous human BChE of genotype Silent (p.Val204Asp)/p.Asp70Gly-p.Ala539Thr (Proband), Silent (p.Val204Asp)/Usual (father) and Usual/p.Asp70Gly-p.Ala539Thr (mother).

**Table 3:** Activity, dibucaine (DN) and fluoride (FN) inhibition numbers of family member’s butyrylcholinesterase (average values from triplicate measurements ± standard errors).

Individual	BChE genotype	Abbreviation	Activity with BTC* (U/mL)	Activity with BzCh° (U/mL)	DN	FN
II-1	p.Val204Asp/p.Asp70Gly-p.Ala539Thr	AKS	0.41±0.02	0.16±0.002	7.2±6.9	15.7±3.7
I-1	p. Val204Asp/Usual	US	2.26±0.13	0.61±0.01	75.3±2.3	51.6±5.0
I-2	p.Asp70Gly-p.Ala539Tyr/Usual	AKU	2.51±0.10	0.63±0.01	58.3±3.6	42.6±4.0
Control #1	Usual/Usual	UU	2.93±0.17	0.71±0.04	78.8±5.9	52.1±5.5
Control #2	p.Asp70Gly/p.Asp70Gly	AA	1.16±0.001	0.39±0.02	5.1±7.6	13.0±4.1
Control #3	p.Asp70Gly/Silent	AS	0.32±0.17	0.12±0.001	11.1±5.6	15.0±2.1

*) 1mM; °) 50 µM.

BChE genotype abbreviations are A for atypical (D70G), K for Kalow variant (A539T), U for usual, S for silent. The proband is II-1; his father is I-1; his mother is I-2.

Hydrolysis kinetics of BTC were performed under steady-state conditions. The catalytic behavior of the patient’s BChE (p.Val204Asp/p.Asp70Gly-p.Ala539Thr) follows the Michaelis-Menten model (boxed reactions in Scheme 1). K_m_ was about 10 times higher than that of the Usual enzyme (18±5 µM) and two times that of the atypical enzyme (150±50 µM) (Control #2 in Table 4). The catalytic behavior of the parent’s BChE fits with expected values for heterozygous enzyme tetramers containing half of usual (U) subunits. The low K_ss_ of the patient’s mother can be explained by the reduced affinity of BTC for the PAS in K-subunits (K_ss_ value of K-BChE was reported to be two-fold lower than Kss of the usual enzyme) [28]. According to Eq 1, the two-fold reduced K_ss_ did not significantly affect kinetic measurements at BTC concentrations lower than 10 mM, i.e. performed at 1mM and 7.56 mM.

**Table 4:** Catalytic properties of patient and parents’s butyrylcholinesterase with BTC as the substrate at pH 7.0 and 25°C (average values from duplicate measurements ± standard deviation).

		BTC			
Individual	BChE genotype	V_max_ (U/ml)	K_m_ (µM)	K_ss_ (mM)	b*
II-1	p.Val204Asp/p.Asp70Gly-p.Ala539Thr	0.50±0.01	265±25	-	1.0
I-1	p. Val204Asp/Usual	7.51±0.01	21±2	0.49±0.08	2.4±0.2
I-2	p.Asp70Gly-p.Ala539Tyr/Usual	6.07±0.01	27±2	0.51±0.02	2.5±0.7
Control #1	Usual/Usual	−(a)	18±5(a)	1.0±0.1(a)	3.2±0.1(a)
Control #2	p.Asp70Gly/p.Asp70Gly	−(b)	150±50(b)	1.1 (b)	1.2±0.2(b)

litterature values [12]: a and b) k_cat_ = V_max_/[E] = 24 000±10 000 min^−1^; b*: substrate activation factor (see Eq 1).

**Figure 3:**
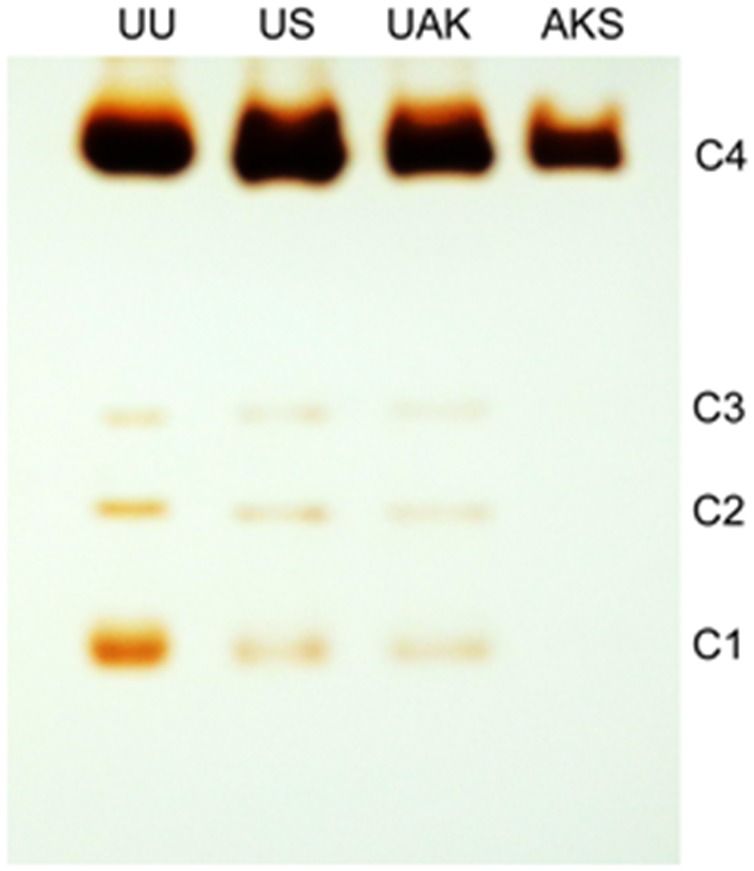
Activity stained polyacrylamide gels of plasma BChE samples. Nondenaturing gel stained for BChE activity. Plasma samples (5 µl per lane) were genotype UU, wild-type usual control; genotype US, the father (I-1) heterozygous for the silent variant; genotype UAK, the mother (I-2) heterozygous for the atypical/K variant; and genotype AKS, the son heterozygous (II-1) for the atypical/K variant and silent BChE. The majority of the plasma BChE is a tetramer (C4). Minor activity bands are for monomer (C1), a BChE monomer- albumin dimer (C2), and a BChE dimer (C3). The AKS plasma has weaker staining intensity for tetrameric BChE (C4) and no apparent minor bands of BChE activity.

**Figure 4:**
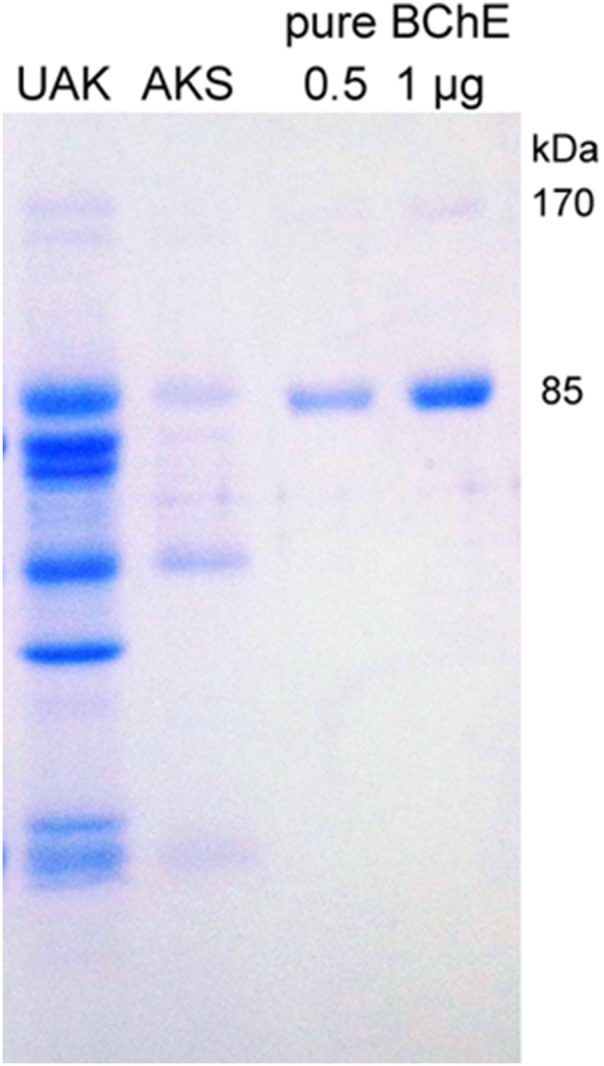
Coomassie Brilliant Blue stained SDS-electrophoresis gel of immunopurified plasma BChE of patient (p.Val204Asp/p.Asp70Gly. Ala539Thr; designated AKS) and his mother (p.Asp70Gly-Ala539Thr; designated UAK). Pure human BChE has a monomer band at 85 µg BChE protein was immunopurified from 0.5 ml plasma of genotype UAK, but only 0.2 µg BChE protein from 0.5 ml plasma of genotype AKS.

Kinetic analysis of the patient’s BChE with BTC as the substrate showed that there was no substrate activation (b = 1), see Individual II-1 in Table 4. This fact indicates that the PAS is not functional in p.Val204Asp subunits or that subunits carrying the p.Val204Asp mutation are totally inactive at all BTC concentrations. Because the tetrameric enzyme is a hybrid of silent subunits carrying the mutation p.Val204Asp and subunits carrying the two mutations p.Gly70Asp and p.Ala539Thr, V_max_ was expected to be reduced compared to parent’s BChE (I-1, I-2) and control BChEs (#1,2). However, V_max_ in the patient’s BChE was unexpectedly low (Tables 3 and 4). This result, was corroborated by electrophoresis data on both activity stained gels and Coomassie Brilliant Blue stained gels of highly purified BChE (Figs. 3 and 4), which indicated that the concentration of BChE in the patient’s plasma is lower than the average BChE concentration of 50 nM. This suggests that mutation p.Val204Asp may either reduce the biosynthesis of enzyme or more likely destabilizes the enzyme and accelerates its clearance from the bloodstream. If the latter possibility is correct this would suggest that mutation p.Val204Asp alters the 3D structure of silent subunits. The MD study on p.Val204Asp mutant protein sheds light on this issue.

An additional cause for the low enzyme concentration could be the p.Ala539Thr mutation. Residue Ala539 is involved in the tetramerization domain of BChE [29]. Mutation p.Ala539Thr (K variant) was found to destabilize the enzyme structure [30]. Therefore the presence of the K variant in subunits carrying the p.Asp70Gly mutation may have also contributed to the low enzyme concentration in the patient’s plasma.

**Figure 5:**
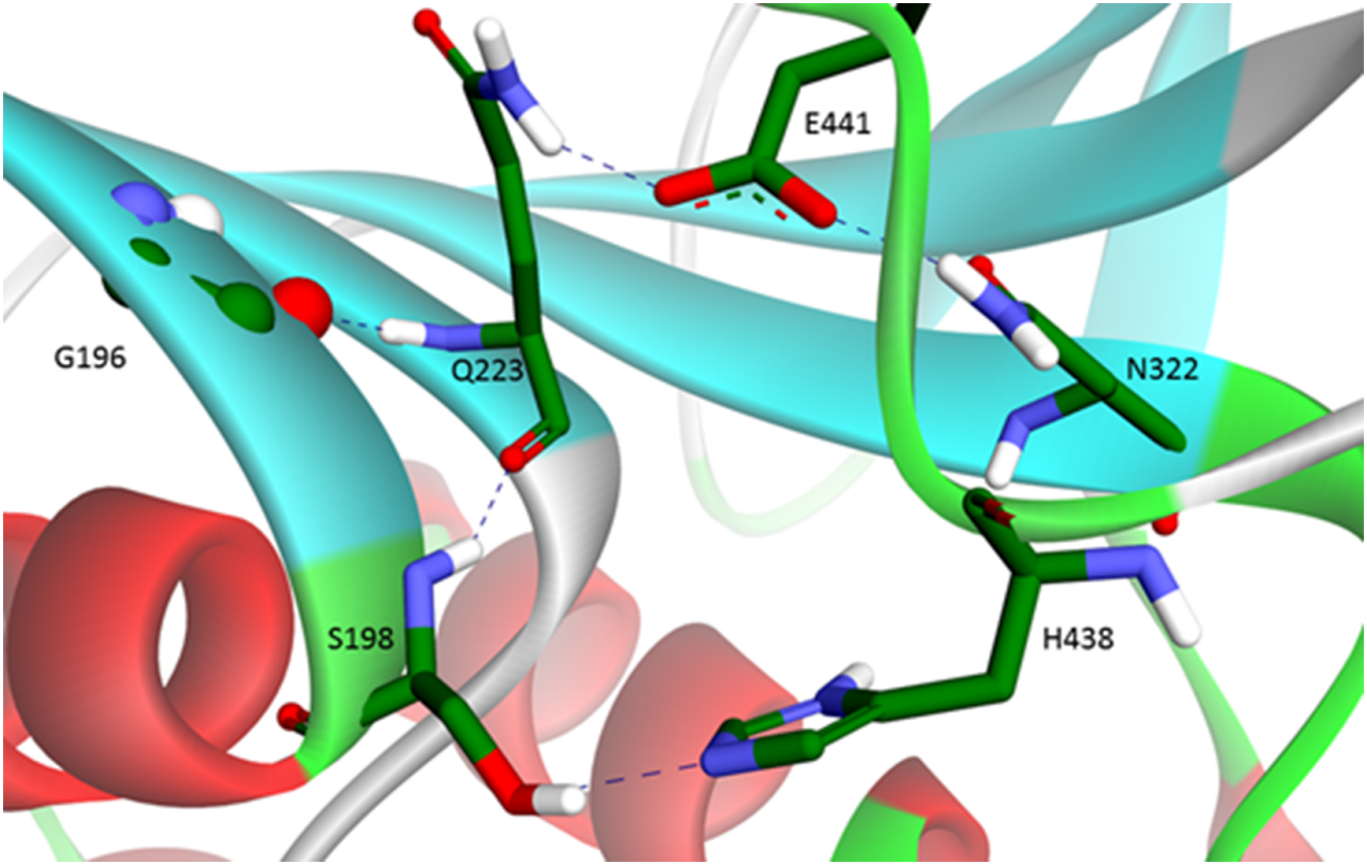
Hydrogen bonding network in usual BChE keeps the α/β-unit turn carrying the catalytic Ser198 close to the loop carrying the catalytic His438. Strands of the β-sheet are fixed together among other hydrogen bonds by ones formed by backbone atoms of Gln223 on one strand, and Gly196 and Ser198 on the other. Side chain of Gln223 forms hydrogen bond with Glu441, this hydrogen bond is maintained during the MD trajectory for usual BChE. As Glu441 is located on the same loop with the catalytic His438, this hydrogen bond is essential for keeping Ser198 and His438 close enough to form functional catalytic triad.

**Figure 6:**
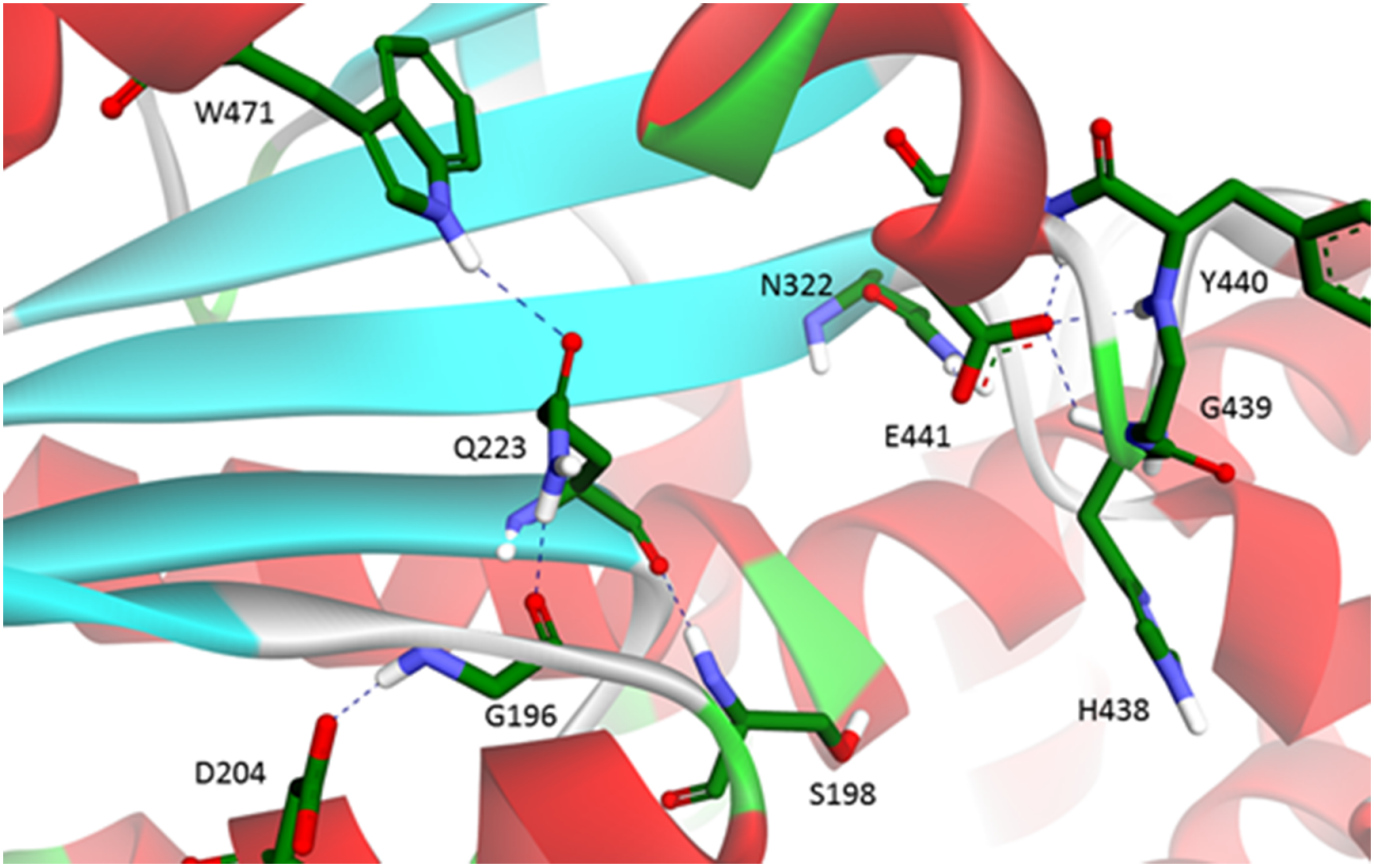
Hydrogen bonding network in p.Val204Asp BChE formed during MD simulation. Histidine 438 faces away from Serine 198, thus disrupting the function of the catalytic triad. This conformational change explains why mutation V204D causes the BChE enzyme to lose activity.

**Figure 7:**
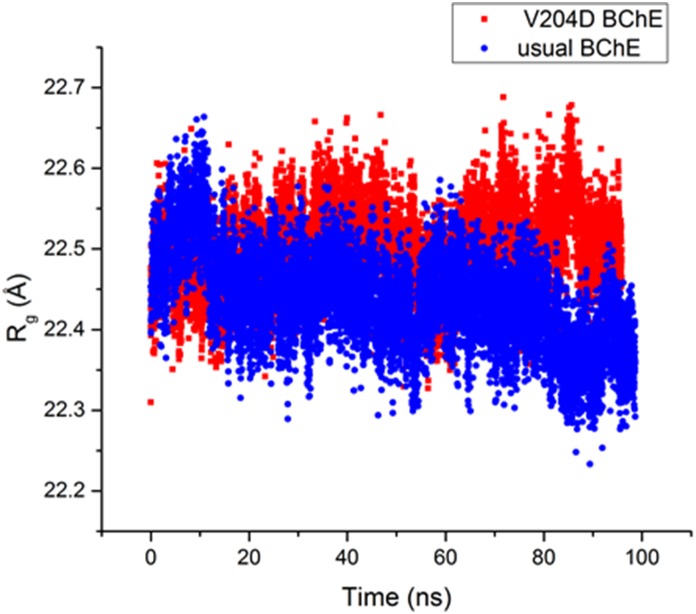
Radius of gyration of BChE in p.Val204Asp mutant and the usual enzyme. Along the MD trajectory size of usual BChE is maintained at the same level while for the mutant it gradually increases what indicates transition to a molten globule state.

Comparison of MD trajectories for usual BChE and p.Val204Asp mutant showed that in the mutant, shortly after the beginning of simulation, the catalytic triad is disrupted. The side chains of Ser198 and His438 move too far from each other to perform catalysis. By contrast, the catalytic triadstays operative during the whole MD trajectory for usual BChE. In usual BChE these catalytic residues are maintained in close proximity by a network of hydrogen bonds involving Gln223, Glu441 and Asn322, which bring the α/β-unit turn carrying the catalytic Ser198 close to the loop carrying the catalytic His438 (Fig. 5). Introduction of a charged amino acid instead of Val204 results in establishment of new hydrogen bonds and disruption of the initial bonds. Asp204 forms a hydrogen bond with Gly196. This causes Gly196 to turn about 90° out of the β-sheet plane (Fig. 6). As a result the Gly196 oxygen backbone forms a hydrogen bond with the Gln223 side chain, instead of with backbone peptide bonds, as for β-sheet strands in usual BChE (Fig. 5). In addition to partial disruption of the β-sheet, this switches hydrogen bonding of the Gln223 side chain from Glu441 to Trp471. In the usual BChE the hydrogen bond between Gln223 and Glu441 links together the β-sheet and the loop carrying the catalytic His438. For Glu441 new hydrogen bonds are formed in the mutant: 3 hydrogen bonds with peptide bond groups of neighboring residues on the His438 loop (Fig. 6). However, Glu441 still interacts with the Asn322 side chain, as in usual BChE. This likely prevents the protein from unfolding. In other words, the overall effect of the mutation p.Val240Asp is disruption of hydrogen bonding between Gln223 and Glu441. This leads the catalytic Ser198 to move away from the loop carrying the catalytic His438 with subsequent disruption of the functional catalytic triad. Therefore, BChE subunits carrying this mutation have no activity regardless of the type of substrate. This mutation also has an impact on the overall protein structure and causes an increase in the radius of gyration along the MD trajectory of p.Val204Asp BChE compared to the usual BChE (Fig. 7). However it does not cause any significant secondary structure change except for that mentioned above (result not shown). The increased gyration radius suggests that the enzyme is in a pre-molten globule state, susceptible to unfolding. This statement would thus explain why the BChE concentration is reduced in plasma.

## Conclusions

In conclusion, this work explains how a point mutation (Val204Asp) far from the active site leads to a “silent” BChE phenotype where the BChE enzyme has no activity regardless of the type of substrate, and has a structure that is not recognized by a monoclonal to native BChE. MD simulations suggest that the enzyme is in a pre-denaturation state.
